# Potential contribution of spinal interneurons to the etiopathogenesis of amyotrophic lateral sclerosis

**DOI:** 10.3389/fnins.2024.1434404

**Published:** 2024-07-18

**Authors:** Luca Goffin, Damien Lemoine, Frédéric Clotman

**Affiliations:** Université catholique de Louvain, Louvain Institute of Biomolecular Science and Technology, Animal Molecular and Cellular Biology, Louvain-la-Neuve, Belgium

**Keywords:** amyotrophic lateral sclerosis, motor neurons, pre-motor interneurons, spinal cord, non-cell autonomous mechanisms, neurodegenerative disease

## Abstract

Amyotrophic lateral sclerosis (ALS) consists of a group of adult-onset fatal and incurable neurodegenerative disorders characterized by the progressive death of motor neurons (MNs) throughout the central nervous system (CNS). At first, ALS was considered to be an MN disease, caused by cell-autonomous mechanisms acting specifically in MNs. Accordingly, data from ALS patients and ALS animal models revealed alterations in excitability in multiple neuronal populations, including MNs, which were associated with a variety of cellular perturbations such as protein aggregation, ribonucleic acid (RNA) metabolism defects, calcium dyshomeostasis, modified electrophysiological properties, and autophagy malfunctions. However, experimental evidence rapidly demonstrated the involvement of other types of cells, including glial cells, in the etiopathogenesis of ALS through non-cell autonomous mechanisms. Surprisingly, the contribution of pre-motor interneurons (INs), which regulate MN activity and could therefore critically modulate their excitability at the onset or during the progression of the disease, has to date been severely underestimated. In this article, we review in detail how spinal pre-motor INs are affected in ALS and their possible involvement in the etiopathogenesis of the disease.

## Introduction

Amyotrophic lateral sclerosis (ALS), also known as Motor Neurone Disease (MND) or Lou Gehrig's disease in the US, consists of a group of adult-onset fatal and incurable neurodegenerative disorders characterized by the progressive death of both cortical upper motor neurons (UMNs) and of brainstem or spinal lower motor neurons (LMNs), leading to fasciculation, spasticity, muscular atrophy and weakness, and eventually paralysis and respiratory failure (Ravits et al., 2007). In Europe, about 50.000 individuals of middle age are affected by ALS, and about 10.000 people die from ALS each year. Worldwide 450.000 people suffer from this pathology, and 120,000 people are diagnosed with ALS annually (Masrori and Van Damme, 2020). The clinical onset of ALS typically occurs between the ages of 40 and 70, and once diagnosed, the disease progresses rapidly, usually leading to the death of the patient from respiratory failure within 3–5 years (Dhasmana et al., 2022). Approximately 90% of ALS cases, known as sporadic ALS (sALS), occur without a familial history. The etiology of sALS remains largely unknown, but some sALS patients have *de novo* mutations found in familial forms. Indeed, the remaining cases, known as familial ALS (fALS), are linked to heritable mutations. Among the most common mutated genes are *chromosome 9 open reading frame 72* (c9orf72, ≈ 40% of fALS), *superoxide dismutase 1* (SOD1, ≈ 20%), *fused in sarcoma* (FUS, ≈ 4%), *optineurin* (OPTN2, ≈ 4%), and *TARDBP* (TDP-43, ≈ 5%) (Taylor et al., 2016). Interestingly, cytoplasmic inclusions of TDP-43 are found in a large majority (≈95%) of all ALS cases, regardless of familial or sporadic status (Kwong et al., 2007). Although ALS affects people of all ethnicities, the prevailing rates of these mutations and clinical symptoms of the disease vary across ethnic groups (Mesaros et al., 2021).

ALS symptoms can be classified into two categories based on the central nervous system (CNS) region where MN degeneration begins. Bulbar symptoms, such as dysarthria and dysphagia, are caused by the loss of brachial/cervical MNs. Spinal symptoms, such as skeletal muscular atrophy and loss of strength in the limbs, result from spinal MN degeneration. However, in both cases, the disease extends over time, eventually affecting all regions of the CNS. The primary cause of death in ALS is respiratory failure due to alterations in MNs controlling the muscles involved in ventilation (see below). Visceral MNs, which innervate cardiac muscle fibers, smooth muscles of visceral organs, and glands, are rarely affected by the disease. In contrast, the most vulnerable MN types in ALS are somatic MNs that innervate fast-twitch fatigable (FF) muscle fibers (Pun et al., 2002; Wootz et al., 2013; Sharma et al., 2016; Spiller et al., 2016). The differential vulnerability of MN subtypes to ALS can be linked to several intrinsic or extrinsic factors such as soma or dendritic tree size, intracellular calcium dynamics, and excitatory/inhibitory synaptic inputs (Nijssen et al., 2017; Ovsepian et al., 2024).

ALS was initially considered an MN disease, caused by autonomous mechanisms affecting specifically these cells. Thus, the research efforts were initially focused on MN and enabled the uncovering of different intrinsic pathological mechanisms. ALS appears to be caused by changes in excitability in multiple neuronal populations, including MNs (Benedetti et al., 2016; Chenji et al., 2016; Kim et al., 2017; Scekic-Zahirovic et al., 2024), which are caused by or associated with a variety of perturbations such as protein aggregation, ribonucleic acid (RNA) metabolism defects, calcium dyshomeostasis, modified electrophysiological properties, and autophagy malfunctions, among others (Srinivasan and Rajasekaran, 2020). However, although the neuronal expression of mutated genes associated with fALS forms is sufficient to induce symptoms related to ALS (Jaarsma et al., 2008; Wang et al., 2009; Huang et al., 2012; Sharma et al., 2016), experimental evidence rapidly demonstrated the involvement of other cell types through non-cell autonomous mechanisms (reviewed in Crabe et al., 2020; Srinivasan and Rajasekaran, 2020; Van Harten et al., 2021). Accordingly, genetic manipulations of ALS genes in astrocytes (Yamanaka et al., 2008b), oligodendrocytes (Kim et al., 2019), and microglial cells (Boillee et al., 2006; Wang et al., 2009) resulted in changes in the progression of the disease in animal models (Yamanaka et al., 2008a). Cell transplantation and co-culture or chimera studies have confirmed the deleterious contribution of astrocytes (Nagai et al., 2007; Qian et al., 2017; Arredondo et al., 2022) and other glial cells (Clement et al., 2003). Surprisingly, the contribution of brainstem or spinal cord pre-motor interneurons (INs), which regulate the activity of LMNs and could therefore critically modulate their excitability at the beginning or during the progression of the disease, has to date been severely underestimated (Gunes et al., 2020; Van Harten et al., 2021).

## Motor circuitry of the spinal cord and ALS

**Figure 1:**
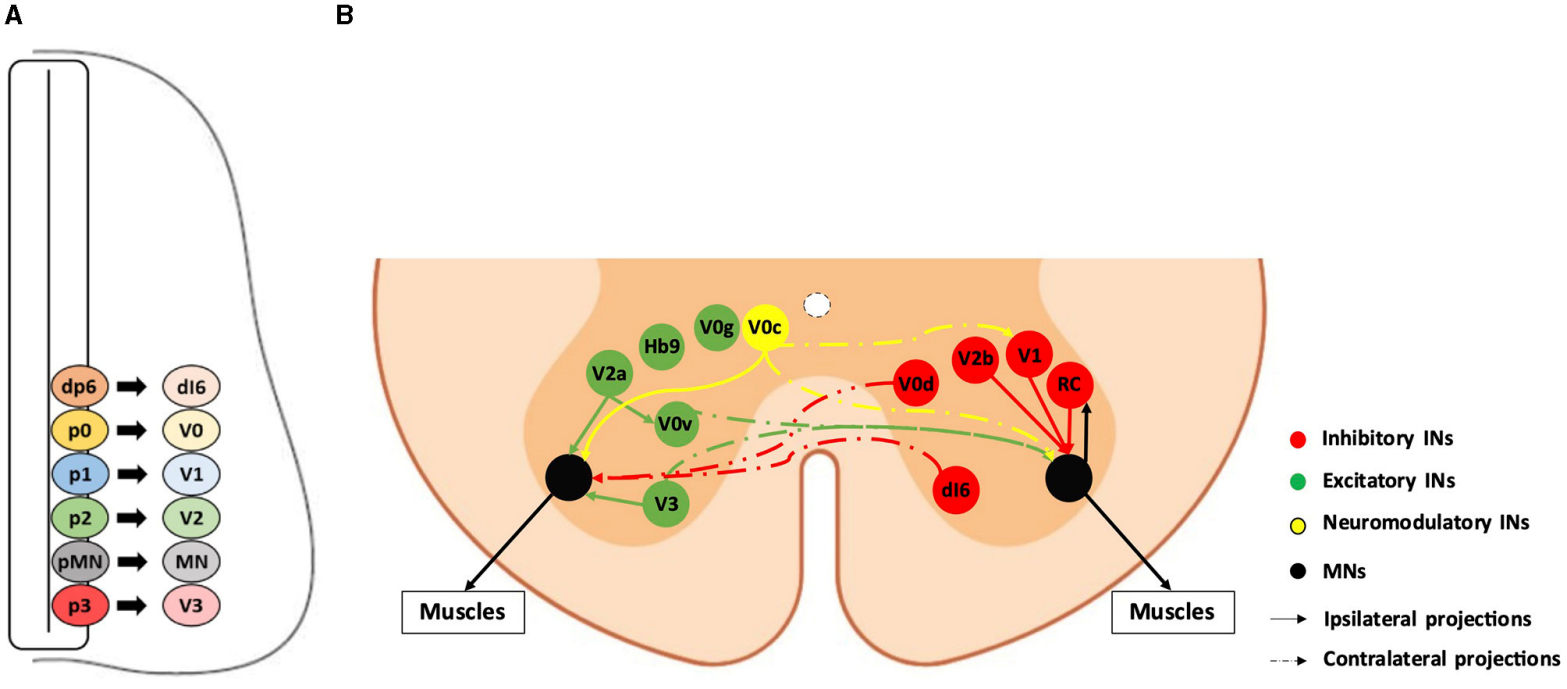
Diversity and connectivity of spinal ventral neuronal populations. A schematic representation of an embryonic transverse hemisection **(A)** or the ventral half of an adult transverse section **(B)** in the spinal cord is shown here. Ventral is to the bottom, dorsal is to the top. **(A)** The six most ventral progenitor domains (dp6, p0, p1, p2, pMN, and p3) and each cardinal neuronal population (dI6, V0, V1, V2, and V3 interneurons [INs], and motor neurons [MN]) arising from their respective progenitor domains. The other half of the spinal cord, as well as the dorsal progenitor domains and neuronal populations, are not represented. **(B)** The MNs and the main pre-motor IN populations involved in the spinal motor circuitry and their known connectivity are represented by colored bubbles (cell body localization) and arrows (projections). For clarity, excitatory or neuromodulatory populations are represented on the left hemicord, and inhibitory populations are represented on the right hemicord, although all these populations are evidently present on both sides. Hb9-IN (the origin of which remains elusive) and V0g connectivity are not precisely known. RC: Renshaw cells (V1 IN subset).

Spinal somatic motor circuits are composed of MNs, which are the effectors of the system and directly control the contraction of skeletal muscles, as well as pre-motor INs, which are connected to each other or directly to MNs and eventually regulate MN activity. Despite numerous limitations in properly defining the diversity of INs in the adult spinal cord (Dougherty, 2023; Roome and Levine, 2023; Dominguez-Bajo and Clotman, 2024), these cells are usually classified according to their embryonic origin and targeted for genetic modifications or lineage-tracing using embryonic-specific markers (Lu et al., 2015). The ventral neural tube contains five IN progenitor domains (pd6, p0, p1, p2, and p3) that produce different pre-motor IN populations (dI6, V0, V1, V2, and V3, respectively) (Figure 1). These cardinal classes of INs have been divided into main populations characterized by unique markers, such as Chx10 for V2a, Gata3 for V2b, Sox1 for V2c, and Shox2 for V2d INs (Wilson and Sweeney, 2023), which can be further subdivided based on the expression of other genes, such as *Sp8, FoxP2, Pou6f2*, or *MafA* for V1 INs or *Onecut* factors *Prdm8, Bhlhb5, MafA*, or *cMaf* for V2 INs (Francius et al., 2013; Bikoff et al., 2016; Gosgnach et al., 2017; Sweeney et al., 2018; Harris et al., 2019). Consequently, subpopulations of INs within the same cardinal class can vary in their location, synaptic targets, activity, and function (Borowska et al., 2013, 2015; Bikoff et al., 2016; Sweeney et al., 2018; Deska-Gauthier et al., 2024). Pre-motor INs regulate MN activity through excitatory, inhibitory, or modulatory synapses. V0v, V0g, V2a, V3, and Hb9-INs are excitatory glutamatergic neurons; V0d, V1, and V2b INs are inhibitory gamma-aminobutyric acid (GABA)ergic/glycinergic neurons; V0c are modulatory cholinergic INs projecting directly on MNs and on V1-derived Ia INs (IaINs) (see below); neurotransmitters used by the other IN subsets have not been identified yet (Figure 1; reviewed among others in Ziskind-Conhaim and Hochman, 2017). At the brachial and lumbar levels, most of these IN populations collectively constitute central pattern generators (CPGs), i.e., the neuronal networks responsible for the general coordination of locomotion (Ramirez-Jarquin and Tapia, 2018). Together, INs and MNs form motor circuitry units within the spinal cord, allowing an accurate activation pattern of spinal MNs for specific motor tasks such as object grasping, body orientation, or locomotion.

A tight balance between the excitation and inhibition levels in motor circuits is crucial to maintaining proper neuronal activity. MN excitotoxicity is one of the mechanisms that contribute to ALS, although the extent of this contribution remains debated (Leroy et al., 2014; Leroy and Zytnicki, 2015). Excitotoxicity, defined as a detrimental response to excitatory stimulations, notably due to poor handling of excessive Ca^2+^, can result from intrinsic hyperresponsiveness of the postsynaptic neuron to physiological glutamate levels or from extrinsic factors including increased extracellular synaptic glutamate concentrations or presynaptic excitation/inhibition imbalance (Van Den Bosch et al., 2006; King et al., 2016). In ALS mouse or zebrafish models, prenatal or postnatal MNs display hyperexcitability in synaptic and firing activities (van Zundert et al., 2008; Martin et al., 2013; Leroy et al., 2014; Benedetti et al., 2016; Jiang et al., 2017) and are intrinsically more responsive to stimulation (Jensen et al., 2020), although hypoexcitability of an MN subset has also been reported (Filipchuk et al., 2021). These defects could be amplified by the alteration of synaptic glutamate recapture by astrocytes via excitatory amino acid transporter 2 (EAAT2, also named glutamate transporter 1 or GLT1) dysfunction (Rosenblum and Trotti, 2017), suggesting a significant contribution of astrocytes to excitotoxicity. However, despite evidence that this mechanism participates in disease progression, inhibition of glutamate uptake alone is not sufficient to recapitulate spinal MN degeneration (Tovar-Y-Romo et al., 2009; Yin and Weiss, 2012). Furthermore, restoration of the glutamate retake function using GLT1 overexpression does not protect mice expressing human *sod1* carrying the G93A mutation found in fALS forms from motor impairment and premature death or delay disease progression (Li et al., 2015). Together, these findings suggest that glutamate transport alterations worsen disease progression in already weakened MNs but fail to explain disease onset.

In parallel to cell-autonomous changes that may modify the excitability of spinal MNs and to the extrinsic contribution of glial cells to these modifications, alterations of pre-motor INs could modulate MN excitability in a non-cell autonomous manner and thereby contribute to the onset or progression of ALS (Morrison et al., 1998; Stephens et al., 2006; Jiang et al., 2009; Hossaini et al., 2011; Benedetti et al., 2016; Qian et al., 2017; Quinlan et al., 2017; Brownstone and Lancelin, 2018). Consistently, in different animal models, INs have been reported to show alterations before MN degeneration in the course of the disease (Niessen et al., 2007; Chang and Martin, 2009; Martin and Chang, 2012; Casas et al., 2013; McGown et al., 2013; Gallart-Palau et al., 2014; Milan et al., 2015; Medelin et al., 2016; Cavarsan et al., 2023). Furthermore, a genome-wide association study (GWAS) in patients provides evidence for cell-autonomous pathology initiation in glutamatergic neurons (van Rheenen et al., 2021), potentially including excitatory spinal INs and resulting in subsequent alterations of spinal MN activity. Consistently, transplanting spinal neural progenitors derived from human sALS-induced pluripotent stem cells in the spinal cord of severe combined immunodeficient (SCID) mice results in endogenous IN alterations that precede MN loss (Qian et al., 2017), suggesting that spinal INs may be more vulnerable than MNs in ALS. Alternatively, the study of a rat model with *TARDBP* overexpression indicated that some IN populations may exert a protective action against MN degeneration (Huang et al., 2012). In this article, we will review in detail how spinal pre-motor INs are affected in ALS and their possible involvement in the etiopathogenesis of the disease.

## Alterations of spinal inhibitory pre-motor INs

**Figure 2:**
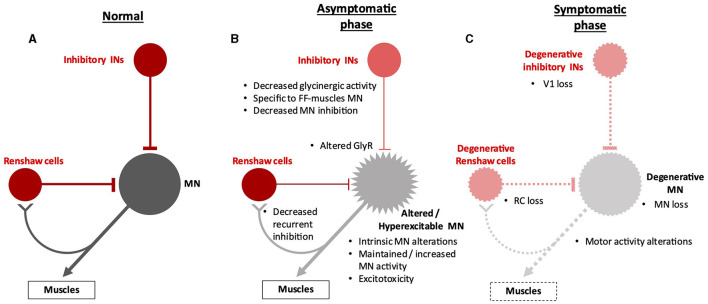
Spinal neuronal inhibitory circuitry and its fate in ALS. Inhibitory interneurons (INs) are represented in red, and motor neurons (MNs) in gray. Altered INs and MNs are represented in light red and light gray, respectively. **(A)** Inhibitory circuitry in a non-pathological situation. **(B)** Early alteration of the inhibitory circuitry before the apparition of the first symptoms. The decrease in glycinergic signaling and in the recurrent inhibition loop ensured by Renshaw cells might act as compensatory mechanisms that preserve motor activity despite low-noise early MN alterations before deleterious breakdown of the circuitry. This possibly results in MN hyperexcitability, represented by spikes on the cell body. This hyperexcitability may contribute to MN excitotoxicity. Panel B summarizes in a single scheme the different alterations involving spinal inhibitory INs, including Renshaw cells, whatever their chronology (for more details, see the proposed working hypothesis in the last section of this article). **(C)** Alterations of the inhibitory circuitry during the symptomatic phase of the disease. Degenerative cells are represented by dotted lines on the cell body and projections. It is still unclear whether degeneration of MNs occurs before or after degeneration of inhibitory INs and if one is causative of the other. RC, Renshaw cells.

Spinal MN activity is tightly dampened by multiple pre-motor GABAergic and/or glycinergic inhibitory IN populations (Figure 1). Numerous studies have reported modifications in GABAergic/glycinergic signaling in the context of ALS. In transgenic SOD1^G93A^ mice, the inhibitory V1 INs (derived from embryonic Engrailed1-positive cells) are reduced in number from the early presymptomatic stages of the disease (Salamatina et al., 2020; Allodi et al., 2021; Montanana-Rosell et al., 2024). The V1 cardinal population contains IaINs involved in the alternation of flexor- or extensor-innervating MN activity during movement (Zhang et al., 2014; Britz et al., 2015), Renshaw cells, which ensure recurrent inhibition on MNs (Alvarez et al., 2005), and other yet unidentified populations. Each V1 IN subset is not equally affected in ALS, suggesting that V1 inhibitory neurons fall into ALS-susceptible or ALS-resistant subpopulations (Salamatina et al., 2020; Montanana-Rosell et al., 2024). This finding is in line with previous results obtained with the same ALS mouse model showing that the number of Renshaw cells is significantly reduced only at the late stages of the disease (Chang and Martin, 2009; Wootz et al., 2013). However, the later degeneration of Renshaw cells may be secondary to the loss of cholinergic input from MNs that parallels MN degeneration (Casas et al., 2013; Wootz et al., 2013). Reduction in neuronal activity of the V1 INs results in a slowing of the motor speed similar to that observed in the early stages of ALS progression in SOD1^G93A^ mice (Gosgnach et al., 2006; Salamatina et al., 2020), suggesting that V1 alterations could be causal to the onset of the disease (Figure 2). Consistently, the presynaptic organizer *Extended Synaptotagmin 1* (*Esyt1*) is downregulated early in V1 INs, and its viral delivery in the spinal cord or overexpression in V1 INs restored V1-MN connectivity, reduced the onset of motor impairment, increased MN survival, and ameliorated motor phenotype (Mora et al., 2024). In contrast, dampening of the V1 activity after the onset of symptoms does not change the SOD1^G93A^ locomotor phenotype (Allodi et al., 2021), indicating that subsequent V1 alterations may not further contribute to the progression of the pathology.

V1 INs establish GABAergic and glycinergic synapses on MNs. Glycinergic synapses are more abundant on MNs innervating fast muscle fibers (fast MNs) than on slow MNs, and V1 has stronger inputs on fast MNs than on slow MNs (Allodi et al., 2021). Interestingly, fast MNs are more affected than slow MNs during ALS progression (see above). Furthermore, SOD1^G93A^ mice or zebrafish display a selective loss of glycinergic inputs on fast MNs before MN degeneration (Hayashi et al., 1981; Pun et al., 2006; Chang and Martin, 2011a,b; Hossaini et al., 2011; McGown et al., 2013; Medelin et al., 2016; Spiller et al., 2016), and global V1 IN input on fast MNs is selectively reduced before symptoms (Allodi et al., 2021). Since other inhibitory IN populations, including V0d and V2b (Figure 1), are not reported to be affected at these early stages of the disease, these observations strongly suggest that this early loss of glycinergic inputs on fast MNs originates at least partly from V1 INs (Figure 2). This global reduction in glycinergic inputs seems greater than the loss of V1 INs (Allodi et al., 2021), which can be explained either by glycinergic synapse disruptions occurring partially without V1 cell death and/or by the possible implication of other glycinergic INs that are yet to be identified. Interestingly, in the early phase of the disease, the number of V1 synapses on stressed or dying MNs is transiently increased, suggesting a possible compensatory mechanism that may be established as an attempt to counteract the hyperactivity of the affected MNs (Wootz et al., 2013; Salamatina et al., 2020). Consistently, a prolongation of inhibitory synaptic events has been observed in fetal SOD1^G93A^ MNs (Branchereau et al., 2019).

In contrast to glycinergic inputs, spinal alterations of GABAergic synapses have not been reported in ALS animal models (Chang and Martin, 2009; Martin and Chang, 2012; Ramirez-Jarquin and Tapia, 2018), although *in situ* hybridization or high-resolution magnetic resonance spectroscopy studies suggested a steady decrease in GABA in the spinal cord from presymptomatic to late disease stages (Niessen et al., 2007; Hossaini et al., 2011), and cortical GABAergic inhibition is reduced in different animal models (Nieto-Gonzalez et al., 2011; Clark et al., 2018) and in human patients (Vucic et al., 2009). Of note, in SOD1^G93A^ mice, rescuing this altered cortical inhibition by activating inhibitory INs delays the onset of the disease (Khademullah et al., 2020). However, functionality and expression of GABAA receptors are altered in SOD1^G93A^ spinal MNs, inducing a higher Cl^−^ influx into the cell with a possible, although counterintuitive, consequent acceleration of neuronal excitotoxicity (Carunchio et al., 2008). Cl^−^ homeostasis alterations were even observed in SOD1^G93A^ prenatal spinal MNs, along with reduced GABAergic/glycinergic activity, suggesting very early inhibitory impairments (Branchereau et al., 2019). Furthermore, organotypic cultures from SOD1^G93A^ spinal cords evidenced that GABA co-release may speed the decay of glycine responses, supported by the detection of a longer persistence of GABA in glycinergic terminals (Medelin et al., 2016). Thus, possible alterations of GABA-dependent inhibition or interactions between GABAergic and glycinergic signaling could contribute to perturbed MN excitability in the onset or progression of ALS (Figure 2), which remains to be investigated in animal models. Furthermore, a more complete characterization of the V1 IN subsets is necessary to provide a more comprehensive understanding of their possible contribution to the pathology. Finally, recent observations reported above highlight the importance of distinguishing different stages of the disease-affected or non-affected neuronal populations in relation to the disease stage and the specificity of each neuronal subtype regarding activity, connectivity, and function.

Recently, several studies investigated the fitness of spinal inhibitory circuitry in human ALS patients using electrostimulation and motor response measuring approaches, notably by assessing Hoffman's reflex (H-reflex) changes. In accordance with the results obtained in animal models, spinal inhibition is reduced in human patients as the disease progresses. Observations suggested that recurrent inhibition involving Renshaw cells (see above) is decreased in patients showing spinal symptoms (Howells et al., 2020; Ozyurt et al., 2020; Sangari et al., 2022; Castro et al., 2024). Interestingly, an increase in recurrent inhibition was observed in the early phase of the disease in one of these studies, supporting the hypothesis of an initial compensatory mechanism preventing MN decline (Sangari et al., 2022). Although conclusions from these studies regarding precise cellular mechanisms, involved IN populations, and timing of occurrence of the different pathological events remain limited, these results are in line with observations in animal models, highlighting their relevance and strongly supporting the hypothesis that spinal interneuronopathy is an important feature of ALS.

Taken together, these data suggest that early changes in inhibitory signaling contribute to the onset of perturbations in spinal MN activity in ALS, possibly resulting in the establishment of transient compensatory mechanisms that may temporarily slow down disease progression before a deleterious breakdown of the motor circuitry (Figure 2).

## Alterations of spinal excitatory pre-motor INs

**Figure 3:**
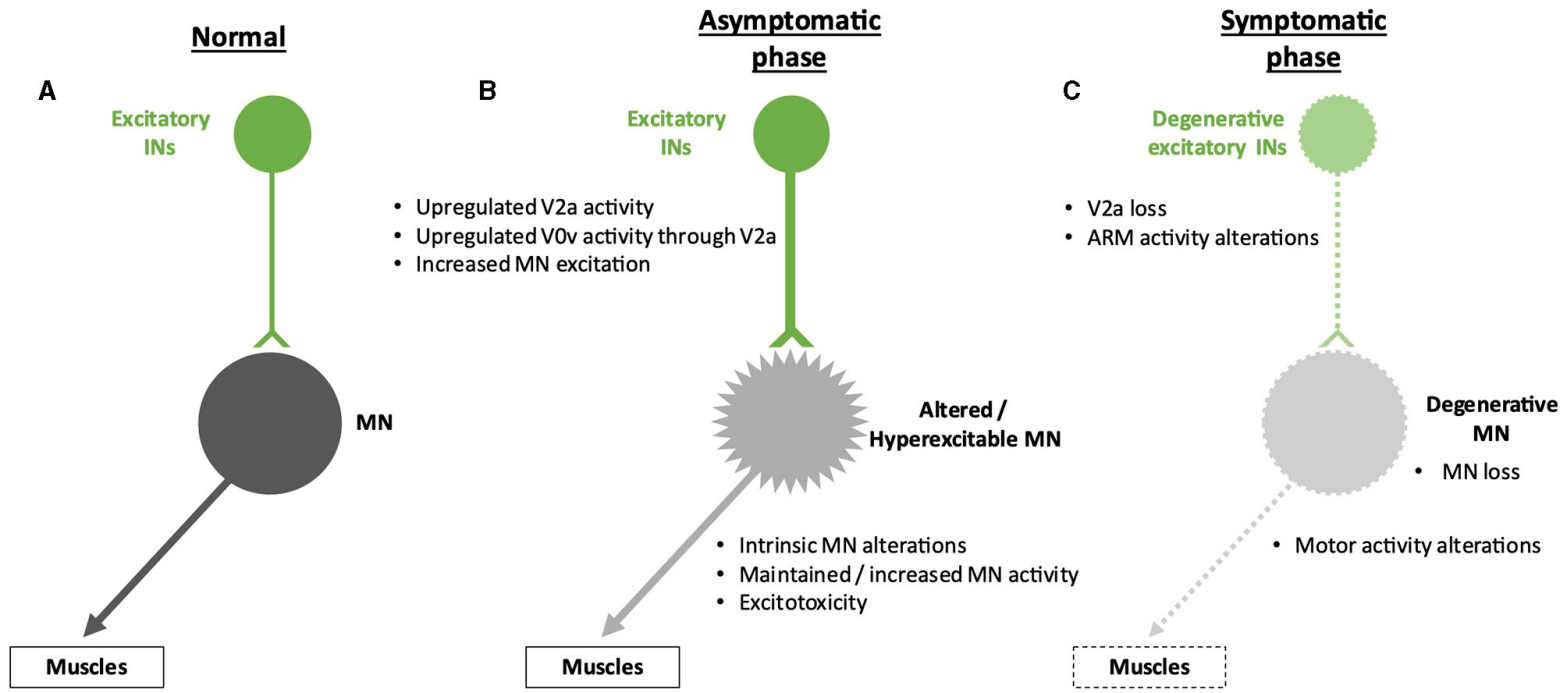
Spinal neuronal excitatory circuitry and its fate in ALS. Excitatory interneurons (INs) are represented in green, and motor neurons (MNs) are represented in gray. Altered INs and MNs are represented in light green and light gray, respectively. Degenerative cells are represented by dotted lines on the cell body and projections. **(A)** Excitatory circuitry in a non-pathological situation. **(B)** Early increase in activity of the excitatory circuitry before the apparition of the first symptoms. This change might preserve motor activity despite low-noise early MN alterations but may result in excitotoxicity for the MNs. The hyperexcitability of the MNs is represented by spikes on the cell body. **(C)** Alterations of the excitatory circuitry during the symptomatic phase of the disease. It is still unclear whether degeneration of MNs occurs before or after degeneration of excitatory INs and if one is causative of the other.

In addition to a reduction in inhibitory inputs, hyperactivation of MNs that can lead to excitotoxicity may result from increased activity of pre-motor excitatory INs. Using an *in vitro* preparation of the sacral cord from SOD1^G93A^ mice, electrophysiology measurements demonstrated that spinal IN activity increases after symptom onset, which may contribute to excitotoxicity, an alteration that involves N-methyl-D-aspartate (NMDA) receptors located either on INs or on MNs (Jiang et al., 2009). Consistently, in zebrafish carrying a human *TARDBP* gene (encoding the TDP-43 protein) with a mutation found in fALS forms, optogenetic activation of reticulospinal neurons and of spinal V2a INs was sufficient to elicit ALS-like symptoms characteristic of this animal model, indicating a functional alteration of glutamatergic synapses in the spinal cord (Petel Legare et al., 2019). However, as suggested for inhibitory inputs on MNs (see above), excitatory INs may increase their activity to compensate for the onset of alterations in MN activity and survival (Figure 3). These compensatory changes might also partly explain the maintenance of motor functions despite affected MNs in the earlier stages of the disease. As an example, accessory respiratory muscle (ARM) activity contributes to preserving ventilation despite diaphragm dysfunctions due to respiratory MN loss in different neurodegenerative disorders, including ALS (Johnson and Mitchell, 2013), and recruitment of the ARM is increased in SOD1^G93A^ mice (Romer et al., 2017). Interestingly, V2a INs innervate ARM MNs and are sufficient to drive increased ARM activity at rest in healthy mice (Romer et al., 2017). Therefore, increased V2a activity may contribute to compensating for the reduced activity of the phrenic MNs in the early stages of ALS. However, V2a INs themselves degenerate in the course of the disease, in parallel with MN degeneration (Romer et al., 2017; Salamatina et al., 2020; Montanana-Rosell et al., 2024). Thus, loss of V2a INs during the progression of the disease will progressively erode the initially activated compensatory mechanism and eventually lead to respiratory failure, the cause of death in most of the ALS cases (Figure 3).

Surprisingly, the contribution of other spinal excitatory INs to the onset or progression of ALS has currently not been addressed. For example, glutamatergic commissural V0v INs are part of an excitatory V2a-V0v circuitry involved in left-right alternation during locomotion (Figure 1) (Lanuza et al., 2004; Crone et al., 2008; Talpalar et al., 2013; Shevtsova, 2015; Ramirez-Jarquin and Tapia, 2018), but the possible contribution of V0v to V2a or MN alterations in ALS has not been reported yet. Similarly, spinal V3 INs contribute to left-right coordination and the robustness of locomotor circuit activity (Figure 1) (Zhang et al., 2008, 2022; Danner et al., 2019), yet their involvement and fate in ALS remain unknown.

## Alterations of spinal cholinergic pre-motor INs

In the brainstem and spinal cord, in addition to inhibitory or excitatory synapses, MNs also receive neuromodulatory inputs. Among these, large cholinergic synapses called C-boutons modulate MN activity by increasing their excitability through m2-muscarinic receptor activation (Miles et al., 2007). Interestingly, C-boutons are found predominantly on fast MNs, the most affected in ALS compared to slow MNs (Hellstrom et al., 2003). A subset of spinal V0 INs, characterized by the expression of the *Pitx2* transcription factor and called cholinergic V0 or V0c (Figure 1), has been identified as the sole source of C-boutons onto MNs (Zagoraiou et al., 2009; Siembab et al., 2010). Of note, V0c also innervate inhibitory V1 IaINs and could therefore indirectly modulate inhibitory inputs on MNs (Siembab et al., 2010).

**Figure 4:**
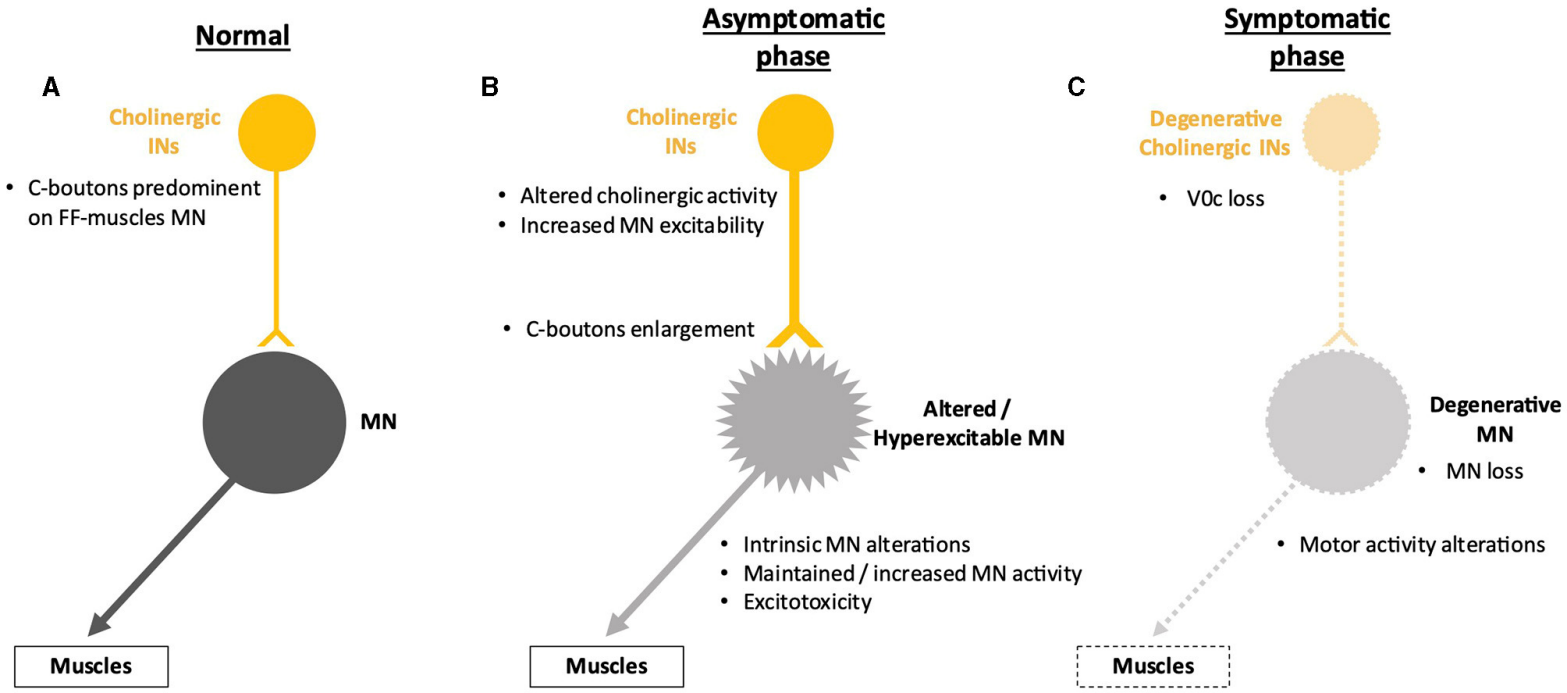
Spinal neuronal cholinergic circuitry and its fate in ALS. Cholinergic interneurons (INs) are represented in yellow, and motor neurons (MNs) in gray. Altered INs and MNs are represented in light yellow and light gray, respectively. Degenerative cells are represented by dotted lines on the cell body and projections. **(A)** Cholinergic circuitry in a non-pathological situation. **(B)** Early increase in activity of the cholinergic circuitry before the apparition of the first symptoms. This modified activity might preserve motor activity despite low-noise early MN alterations but may result in excitotoxicity for the MNs. The hyperexcitability of the MNs is represented by spikes on the cell body. This hyperexcitability may contribute to MN excitotoxicity. **(C)** Alterations of the cholinergic circuitry during the symptomatic phase of the disease. It is still unclear whether degeneration of MN occurs before or after degeneration of cholinergic INs and if one is causative of the other.

Changes in C-boutons in ALS have been observed both in humans (Nagao et al., 1998) and in mouse models (Chang and Martin, 2009; Pullen and Athanasiou, 2009; Herron and Miles, 2012; Gallart-Palau et al., 2014; Milan et al., 2015). However, it remains unclear whether these changes are compensatory to early MN alterations or intrinsic to V0c INs (Brownstone and Lancelin, 2018). In SOD1 mouse models, the density of C-boutons around MN soma was transiently increased at early presymptomatic stages (Herron and Miles, 2012; Gallart-Palau et al., 2014; Milan et al., 2015), possibly only on slow MNs (Bak et al., 2023). Furthermore, the size of the C-boutons was increased as early as postnatal day 8, long before symptom onset and MN degeneration (Pullen and Athanasiou, 2009; Herron and Miles, 2012; Milan et al., 2015). Interestingly, the persistence of this early change until adult stages was only observed in male SOD1 mice, suggesting a gender effect on cholinergic alterations in ALS (Herron and Miles, 2012). A similar gender bias has been reported in a mutated *TARDBP*-based ALS mouse model regarding changes in C-bouton numbers on fast or slow MNs (Bak et al., 2023). These observations could relate to the higher prevalence of ALS in men than in women (Zamani et al., 2024). Given that C-boutons increase MN excitability (Zagoraiou et al., 2009; Witts et al., 2014; Nascimento et al., 2020; Wells et al., 2021), an early increase in density and enlargement could contribute to MN hyperexcitability and participate in excitotoxic mechanisms leading to MN degeneration (Figure 4). However, in the same model, ChAT levels are shown to be reduced in MNs, V0c INs, and C-boutons in the presymptomatic phase (Casas et al., 2013). This suggests that enlargement of C-boutons could compensate for early cholinergic alterations in MNs or at C-boutons rather than being causative of MN hyperexcitability. Consistently, C-boutons activation was increased in low-intensity tasks like walking in SOD1^G93A^ mice as compared to wild-type mice (Wells et al., 2021). This rescue hypothesis was tested by genetically inactivating C-boutons in the SOD1 mouse model, but studies reported opposite results. In the earlier study, C-bouton inactivation resulted in an acceleration in the apparition of the disease phenotype (Landoni et al., 2019), suggesting that these cholinergic synapses may protect MN against early alterations (Wells et al., 2021), possibly through C-bouton enlargement. This compensation of motor impairments by V0c would decrease as the C-boutons are lost at later stages of the disease (Nagao et al., 1998; Pullen and Athanasiou, 2009; Casas et al., 2013; Bak et al., 2023). In later studies, mice performed slightly better in ALS-related motor tasks, and innervation of fast-twitch muscles was longer preserved after C-bouton inactivation, although without any change in the “humane endpoint,” suggesting a deleterious effect of cholinergic inputs early during disease progression (Konsolaki et al., 2020; Wells et al., 2021). In line with this finding, deletion of TMEM16F, a Ca^2+^-activated Cl^−^ channel present at the postsynaptic face of C-boutons and involved in neuromodulation of MN activity, delays disease onset in SOD1^G93A^ mice, again only in male mice (Soulard et al., 2020). Thus, modifications in C-bouton numbers and size may initially compensate for early alterations in MN fitness but may later become detrimental for MN innervation and survival, resulting in defective motor activity (Figure 4).

In any case, despite contradictory observations on C-bouton fate and contribution to MN degeneration in ALS, all these studies highlighted early presymptomatic alterations of cholinergic inputs on MNs before MN degeneration occurs. Although their number is transiently increased at these early stages (Herron and Miles, 2012; Milan et al., 2015; Bak et al., 2023), a later reduction in C-boutons and degeneration of the V0c INs is observed in symptomatic SOD1 or TDP43 mouse models of ALS (Chang and Martin, 2009; Casas et al., 2013; Milan et al., 2015; Wells et al., 2021; Bak et al., 2023). The reduction in C-boutons in the symptomatic phase of the disease may result from MN degeneration losing its afferent cholinergic synapses (Wells et al., 2021). In line with this hypothesis, *Neuregulin-1* expression, a factor present at the postsynaptic face of C-boutons, is diminished in MNs of an SOD1 mouse model and of ALS patients (Gallart-Palau et al., 2014; Lasiene et al., 2016), and its administration restores C-bouton numbers in mice (Lasiene et al., 2016). Whether the subsequent loss of V0c INs is a consequence of MN degeneration or results from intrinsic alterations that parallel MN defects remains to be investigated.

Taken together, although alterations of C-boutons and V0c INs in ALS have been demonstrated, a more systematic investigation of each alteration at each stage of disease progression in each available model should be carefully conducted to identify the involved cellular and molecular mechanisms, possible gender bias, whether these changes are beneficial or detrimental for ALS onset or progression, and how much these could constitute potential therapeutic targets to slow down the progression of the pathology. This said, it is plausible that the contribution of C-boutons is not strictly protective or deleterious but may differ depending on the stage of the disease. An increase in V0c cholinergic inputs on MNs in the presymptomatic phase discussed above might initially occur as an early effort to preserve motor unit activity by increasing MN excitability. However, this increased MN excitability, along with weakened MN and altered excitation/inhibition balance (see above), could eventually be detrimental to MN integrity through excitotoxic mechanisms and thus participate in their degeneration (Figure 4).

## Conclusion and perspectives

ALS is a complex disease that encompasses several intrinsic and extrinsic mechanisms leading to MN death, inevitably affecting the whole motor control circuitry. In such a context, the proof of a single pathological trigger is still missing, and its very existence might be questioned. A more plausible hypothesis would favor a combination of multiple pathological events resulting in MN degeneration.

An accurate illustration of this conclusion resides in the involvement of spinal INs in the disease course. In the spinal cord motor circuitry, excitatory, inhibitory, or cholinergic INs are tightly regulating MN activity and excitability by establishing a versatile but well-controlled excitation/inhibition balance. Alterations in IN populations observed in ALS are deregulating this balance and thus most likely contribute to MN degeneration, notably through excitotoxic mechanisms. Based on the publications reviewed above, a working hypothesis can be proposed. Each type of spinal IN, i.e., inhibitory, excitatory, or cholinergic INs, shows changes in the number or activity of the synapses they form on MNs before the onset of MN degeneration or symptom apparition, either as a result of intrinsic alterations or as an attempt to compensate for early low-noise perturbations in MN activity. These early changes, although transiently preserving the fitness of the motor circuitry, may progressively result in the hyperexcitability of MNs, which are weakened by intrinsic alterations or other non-cell autonomous mechanisms. Even though this hyperexcitability has also been described as a possible protective mechanism (Leroy and Zytnicki, 2015; Huh et al., 2021), it would eventually lead to excitotoxicity and MN degeneration, possibly causing parallel degeneration of the pre-motor IN populations (Figures 2–4). The currently available therapy for ALS, a glutamate antagonist that reduces excitotoxicity, may transiently slow down MN degeneration caused by a combination of these mechanisms but becomes rapidly inefficient to counteract the general collapse of all the components of the spinal motor circuitry.

The identification of novel therapeutic options will require a systematic characterization of the contribution of each spinal IN population, alone or in combination with MNs, other INs, or glial cells, to the onset or progression of ALS. Furthermore, forthcoming studies should consider any gender bias, carefully report on the precise disease stage of each analysis, and systematically discriminate the type of motor units that are investigated. However, most of the reported observations have been obtained in SOD1-based animal models of the disease. Importantly, *Sod1* mutations only affect a small fraction of ALS cases in humans, raising questions about the validity of these models to identify general mechanisms of the pathology. In contrast, TDP43-based models recapitulate a feature found in more than 95% of all ALS cases, i.e., cytoplasmic TDP43 aggregation (Suk and Rousseaux, 2020), and might therefore constitute more promising approaches to studying the disease (Spiller et al., 2016).

## Author contributions

LG: Writing – original draft, Writing – review & editing. DL: Writing – review & editing. FC: Writing – original draft, Writing – review & editing.
